# A meta-analysis with systematic review: Efficacy and safety of immune checkpoint inhibitors in patients with advanced gastric cancer

**DOI:** 10.3389/fonc.2022.908026

**Published:** 2022-10-31

**Authors:** Aya El Helali, Jun Tao, Charlene H. L. Wong, Wendy Wing-Lok Chan, Ka-Chun Mok, Wing Fong Wu, Kohei Shitara, Markus Mohler, Narikazu Boku, Herbert Pang, Ka On Lam

**Affiliations:** ^1^ Department of Clinical Oncology, Li Ka Shing Faculty of Medicine, The University of Hong Kong, Queen Mary Hospital, Hong Kong, Hong Kong SAR, China; ^2^ School of Public Health, Li Ka Shing Faculty of Medicine, The University of Hong Kong, Hong Kong, Hong Kong SAR, China; ^3^ Department of Gastrointestinal Oncology, National Cancer Center Hospital East, Kashiwa, Japan; ^4^ Department of Medicine, Johannes-Gutenberg University Clinic, Mainz, Germany; ^5^ Department of Oncology and General Medicine, IMSUT Hospital, Institute of Medical Science, The University of Tokyo, Tokyo, Japan; ^6^ Department of Biostatistics and Bioinformatics, Duke University of Medicine, Durham, NC, United States

**Keywords:** advanced gastric adenocarcinoma, immune checkpoint inhibition (ICI), PD-L1, tumor mutational burden (TMB), microsatellite instability (MSI)

## Abstract

**Background:**

While the efficacy of immune checkpoint inhibitors (ICIs) is increasingly recognized in advanced gastric cancer (aGC), overall survival (OS) has not been consistently improved across the different randomized controlled trials (RCTs). This meta-analysis aimed to quantify the efficacy and safety of ICI and explore potential predictive tumor tissue biomarkers in aGC.

**Methods:**

A random-effect pairwise meta-analysis was used to evaluate the primary outcome of OS. Sensitivity analysis was performed to investigate the effects of ICIs on PD-L1 status, TMB, MSI-H, and the Asian patient population. We extracted the OS Kaplan–Meier curves from the included trials to compare the effect of PD-L1 status on response to ICIs using DigitizeIt 2.5 and Guyot’s algorithm.

**Results:**

A pairwise meta-analysis of seven RCTs included in this study showed that ICIs were more effective than the comparator in improving OS (pooled HR: 0.84). We demonstrated that PD-1 ICIs were additive when combined with the comparator arm (pooled HR: 0.79). A sensitivity analysis showed that PD-1 ICIs were associated with better OS outcomes in the Asian patient population as monotherapy (pooled HR: 0.66) or in combination with chemotherapy (pooled HR: 0.83). We demonstrated that tumors with PD-L1 ≥1 (*P* = 0.02) and PD-L1 ≥10 (*P* = 0.006) derived OS benefit from ICI monotherapy. Equally, MSI-H (*P <*0.00001) and TMB-high (*P <*0.0001) tumors derived favorable survival benefits from ICIs.

**Conclusions and relevance:**

The results of this meta-analysis suggest that ICIs result in improved OS outcomes in aGC. The benefits varied with different ethnicities, class of ICI, PD-L1 expression, MSI status, and TMB

**Systematic Review Registration:**

https://www.crd.york.ac.uk/prospero, identifier (CRD42019137829).

## Introduction

The high mortality rate associated with gastric cancer (GC) (1) highlights the unmet need for novel treatment strategies that harness a personalized approach. Asia accounts for approximately 60% of the world’s current population (2) and has the highest age-standardized incidence of GC (27.3–31.0 per 100,000 population) (3). It is the fourth leading cause of cancer deaths worldwide (1). More importantly, GC mortality rates are highest in eastern Asia (4, 5), representing the second most common cause of death after lung cancer (6) within the region.

Immunotherapy, through the inhibition of immune checkpoints, such as the interaction between the tumor-derived programmed death ligand-1 (PD-L1) and the programmed death-1 (PD-1) receptor on T cells, is a rapidly expanding field for all solid cancers and now plays an essential role in the management of GC (7). However, the clinical efficacy of immune checkpoint inhibitors (ICIs) in GC is marred by a predominantly immune cold or immune-excluded tumor microenvironment, with the exception of the microsatellite instability-high (MSI-H) and Epstein–Barr virus (EBV) molecular subtypes (8). There are controversies regarding the use of an appropriate clinically validated predictive biomarker to select the right patient population that will confer response from ICIs in GC.

Seven phase III randomized controlled trials have evaluated the role of ICIs in managing advanced GC. The clinical efficacy of nivolumab was highlighted in three distinct clinical settings. Firstly, ATTRACTION-2 (9) addressed the role of single-agent nivolumab in managing treatment-refractory GC. It demonstrated a statistically significant improvement in overall survival (OS) in the nivolumab alone group in comparison to the placebo group (hazard ratio (HR) of 0.63; 95% confidence interval (CI): 0.51–0.78; P <0.0001) (9). Conversely, CheckMate-649 (10) validated the clinical efficacy of nivolumab combined with chemotherapy in treatment-naïve advanced GC. There was also a statistically significant improvement in OS among advanced GC patients with a PD-L1 combined positive score (CPS) status of ≥5 (HR: 0.71; 98.4% CI: 0.59–0.86; *P <*0.0001). Importantly, OS was significantly improved in all enrolled patients (HR: 0.80; 99.3% CI: 0.68–0.94; *P* = 0.0002) (10). However, conflicting results were shown in ATTRACTION-04 (11), conducted among Asian patients. It was demonstrated that nivolumab did not improve OS (HR: 0.90; 95% CI 0.75–1.08; *P* = 0.26) in patients with previously untreated, HER2-negative, advanced GC or gastro-esophageal junction (GEJ) cancer (11). In the KEYNOTE-061 trial, treatment with pembrolizumab failed to show a significant survival benefit compared with paclitaxel chemotherapy, based on PD-L1 CPS ≥1 (HR: 0.82; 95% CI: 0.66–1.03; P = 0.042), in patients who have failed first-line treatment (12). In KEYNOTE-062, pembrolizumab monotherapy demonstrated non-inferiority to chemotherapy in patients with chemotherapy naïve PD-L1 CPS ≥1 advanced GC (HR: 0.91; 99.2% CI: 0.69–1.18; non-inferiority margin = 1.2) (13). Similarly, when combined with chemotherapy, pembrolizumab was not superior to chemotherapy (PD-L1 CPS ≥1: HR: 0.85; 95% CI, 0.70–1.03; *P* = 0.05; PD-L1 CPS ≥10: HR: 0.85; 95% CI: 0.62–1.17; *P* = 0.16) (13). Interestingly, pembrolizumab monotherapy resulted in a prolonged OS (HR: 0.69; 95% CI: 0.49–0.97) in patients with a PD-L1 CPS ≥10 (13). The clinical significance of avelumab, an IgG1 monoclonal antibody targeting PD-L1, was evaluated in JAVELIN Gastric 300 and JAVELIN Gastric 100. The JAVELIN Gastric 300 trial failed to demonstrate the clinical efficacy of avelumab in the≥third-line setting (HR: 1.1; 95% CI: 0.9–1.4; *P* = 0.81) (14). This observation was further strengthened in the JAVELIN Gastric 100 trial, in which maintenance avelumab did not demonstrate superior OS both in the overall population (HR: 0.91; 95% CI: 0.74–1.11; *P* = 0.178) and the predefined PD-L1 positive population (HR: 1.13; 95% CI: 0.57–2.23; *P* = 0.635) compared with chemotherapy (15). In most of the trials discussed above, patients with higher PD-L1 expression showed a trend toward better survival following treatment with immune checkpoint inhibitors.

To date, nivolumab has received regulatory approval from the US Food and Drug Administration (FDA) as a first-line therapy in combination with chemotherapy for advanced or metastatic GC, GEJ cancer, and esophageal adenocarcinoma (16). The FDA licensed pembrolizumab to manage recurrent, locally advanced or metastatic gastric or GEJ adenocarcinoma whose tumors express PD-L1 (≥1) following ≥two lines of standard of care chemotherapy (17). In addition, the FDA approved the frontline clinical use of pembrolizumab in combination with trastuzumab, fluoropyrimidine- and platinum-containing chemotherapy in advanced unresectable or metastatic HER2-positive gastric or GEJ adenocarcinoma (18). In Europe, nivolumab has been approved in the first-line setting in patients with advanced or metastatic gastric tumors expressing PD-L1 CPS ≥5.

We identified a need to streamline the trial findings and, therefore, performed a comprehensive meta-analysis to quantify the impact of PD-L1 status, MSI, tumor mutational burden (TMB), and ethnicity on survival outcomes following treatment with ICIs.

## Methods

We performed a systematic review and meta-analysis of the RCTs that compared the efficacy of ICIs as monotherapy or in combination with standard of care treatment, as defined in current, evidence‐based guidelines for systemic therapy, of patients with either locally advanced or metastatic gastric cancer. Our study was conducted according to the Preferred Reporting Items for Systematic Reviews and Meta-Analyses (PRISMA) guidelines (19). A prospective protocol was registered in PROSPERO (CRD42019137829**)** (20). Details of the literature search strategy, data collection, and assessment of study quality are described in the **Supplementary Methods**.

### Data extraction

We established a data abstraction method to effectively collect the necessary information from our search strategy that was pilot-tested ahead of time (see **Supplementary Methods** for details). Furthermore, we received further information to comprehensively conduct the meta-analysis for the ATTRACTION-02 trial from Professor Narikazu Boku and JAVELIN Gastric 300 from Professor Markus Möhler and the EMD/Merck group.

### Statistical analysis

Pairwise meta-analyses for the primary outcome of OS in the intention-to-treat population, tissue biomarkers (PD-L1, MSI-H, and TMB), and safety assessments were performed using RevMan v5.4. A random-effects model was planned for this study. Sensitivity analysis on OS was conducted by focusing on i) the Asian population, ii) PD-L1 expression, iii) MSI-H, and iv) TMB. Subgroup analysis was conducted by stratifying OS into three groups: i) PD-1 ICI monotherapy versus comparator; ii) PD-1 ICI combined with comparator versus comparator; and iii) PD-L1 ICI monotherapy versus comparator. This systematic review defined the comparator as the active treatment in the control arm or delivered in combination with immunotherapy. We used pooled hazard ratios (HRs) with 95% confidence intervals (CIs) to express the primary outcome. However, we reported our findings for the secondary outcome of immunotherapy-related adverse events using a risk ratio. The corresponding forest plots were illustrated. Statistical heterogeneity was evaluated using the Q test and the inconsistency index (I^2^). As per the Cochran definition, Cochran’s Q test is the traditional test for heterogeneity in meta-analyses (21). A P-value of <0.10 indicates significant heterogeneity. I^2^ estimates the percentage of variability in results across studies due to real differences and not due to chance (21). I^2^ values with <25% were regarded as low heterogeneity, 25%–50% as moderate level, and >50% as high level (22).

Using DigitizeIt 2.5 software (https://www.digitizeit.xyz) and Guyot’s algorithm, we extracted the OS survival curves from the trials included in our study. Using this method, we compared the effects of PD-L1 <1, PD-L1 ≥1, and PD-L1 ≥10 status on response to single-agent ICI (23, 24). The Cox regression model stratified by the study was used to model the survival data. P-values for the two individual curves (PD-L1 ≥1 and PD-L1 ≥10) were generated, taking the group of PD-L1 <1 as the reference. The corresponding Kaplan–Meier (KM) survival curves were illustrated.

## Results

### Systematic review and characteristics

A total of 1,047 publications were identified through the initial literature search, and 997 studies remained after duplications were excluded. Following the title and abstract screens, 945 publications were removed because they did not meet this study’s hypothesis or were abstracts of full-text publications included in the eligible article review. Fifty-two potentially relevant articles were identified for the comprehensive review (**Supplementary Figure 1**). Following this process, seven multicenter phase III RCTs with a total number of 5,023 patients (median: 592; range: 371–1,581) were analyzed in this meta-analysis. The primary trial outcomes and trial characteristics are summarized in **Table 1**.

**Table 1:** Characteristics of randomized controlled trials. In the meta-analysis.

Trial Title	Study ID	Immunotherapy Target	Disease Setting	Treatment Sequence	Randomization Criteria	Intervention	Patents Recruited (n)	Patients Recruited per study arm (n)	Median OS [Months (95% CI)]	Hazard Ration (95% CI)	Median PFS [Months (95% CI)]	Hazard Ration (95% CI)	Asian Population	Median OS [Months (95 CI)]	Hazard Ratio (95 CI)
JAVELIN Gastric 300	NCT02625623	PD-L1	Advanced Gastric/Gastro-esophageal Cancer	Third Line Treatment	PD-L1 status not required for randomization	Arm A: avelumab	371	185	4.6 (3.6–5.7)	1.1 (0.9–1.4); P = 0.81	1.4 (1.4–1.5)	1.73 (1.4–2.2); P >0.99	47	5.7	1.25 (0.79-1.98)
						Arm B: Drug: irinotecan Drug: paclitaxel Drug: BSC		186	5.0 (4.5–6.3)		2.7 (1.8–2.8)		47	7	
ATTRACTION-2	NCT02267343	PD-1	Unresectable or recurrent Gastric/Gastro-esophageal Cancer	≥ 2 Prior lines of treatment	PD-L1 status not required for randomization	Arm A: nivolumab	493	330	5.26 (4.60–6.37)	0.63 (0.51–0.78); P <0.0001	1.61 (1.54–2.30)	0.60 (0.49–0.75); P <0.0001	330	5.26 (4.60–6.37)	0.63 (0.51–0.78); P <0.0001
						Arm B: placebo		163	4.14 (93.42–4.86)		1.45 (1.45–1.54)		163	4.14 (93.42–4.86)	
KEYNOTE-061	NCT02370498	PD-1	Locally advanced, unresectable, or metastatic Gastric Cancer	Second-Line	PD-L1 CPS <1; PD-L1 CPS ≥1 (Recruitment restricted to patients with PD-L1 ≥1 after an interim outcome analysis)	Arm A: pembrolizumab	592	296	9.1 (6.2–10.7)	0.82 (0.66–1.03); P = 0.0421	1.5 (1.4–2.0)	1.27 (1.03–1.57)	88	NR	0.90 (0.59–1.38)
						Arm B: paclitaxel		296	8.3 (7.6–9.0)		4.1 (3.1–4.2)		89	NR	
KEYNOTE-062	NCT02494583	PD-1	Locally advanced, unresectable, or metastatic Gastric Cancer	First-Line	PD-L1 CPS ≥1	Arm A: pembrolizumab	763	256	PD-L1 ≥1: 10.6 (7.7–13.8)	PD-L1 ≥1: HR-0.91 (0.69–1.18)	PD-L1 ≥1: 2.0 (1.5–2.8)	PD-L1 ≥1: HR = 1.66 (1.37–2.01)	62	PD-L1 ≥1: 22.7 (14.3–28.5)	PD-L1 ≥1: 0.54 (0.35–0.82)
						Arm B: pembrolizumab Drug: cisplatin Drug: 5-FU Drug: capecitabine		257	PD-L1 ≥1:12.5 (10.8–13.9)	PD-L1 ≥1: HR-0.85 (0.70–1.03); P = 0.05	PD-L1 ≥1: 6.9 (5.7–7.3)	PD-L1 ≥1: HR-0.84 (0.70–1.02); P = 0.04	64	NR	PD-L1 ≥1: 0.78 (0.53–1.16)
						Arm C: Drug: cisplatin Drug: 5-FU Drug: capecitabine Drug: placebo		250	PD-L1 ≥1:11.1 (9.2–12.8) & PD-L1 ≥10: 10.8 (8.5–13.8)		PD-L1 ≥1: 6.4 (5.7–7.0) & PD-L1 ≥10: 6.1 (5.3–6.9)		61	PD-L1 ≥1: 13.8 (10.5–16.9)	
CheckMate 649	NCT02872116	PD-1	Locally advanced, unresectable, or metastatic Gastric Cancer	First-Line	PD-L1 status not required for randomization	Arm A: nivolumab	1,581	789	13.8 (12.6–14.6)	0.8 (99.3 CI: 0.68–0.94); P = 0.0002	7.7	0.77 (0.68–0.87)	186	NR	0.73 (0.57–0.93)
						Arm B: chemotherapy		792	11.6 (10.9–12.5)		6.9		189	NR	
JAVELIN Gastric 100	NCT02625610	PD-L1	Locally advanced, unresectable, or metastatic Gastric Cancer	First-Line maintenance	PD-L1 status not required for randomization	Arm A: avelumab maintenance	499	249	10.4 (9.1–12.0)	0.91 (0.74 to 1.11)	3.2	1.04 (0.85–1.28)	57	10.8 (9.0 to 16.1)	0.90 (0.59–1.36)
						Arm B: chemotherapy maintenance		250	10.9 (9.6–12.4)		4.4		57	11.9 (9.5 to 15.0)	
ATTRACTION-04	NCT02746796	PD-1	Unresectable or recurrent Gastric/Gastro-esophageal Cancer	First-Line	PD-L1 status was required prior to randomization	Arm A: nivolumab plus investigator’s choice of chemotherapy	724	362	17·45 (15·67–20·83)	0·90 (0·75–1·08); P = 0·26	10·94 (8·44–14·03)	HR 0·68 (98·51% CI 0·51–0·90); P = 0·0007	362	17·45 (15·67–20·83)	0·90 (0·75–1·08); P = 0·26
						Arm B: placebo plus chemotherapy		362	17·15 (15·18–19·65)		8·41 (7·03–9·69)		362	17·15 (15·18–19·65)	

PD-L1, programmed cell death ligand 1; PD-1, programmed cell death 1; CPS, combined positive score; ERBB2, human epidermal growth factor receptor 2; OS, overall survival; PFS, progression free survival; ORR, objective response rate; HR, hazard ratio; CI, confidence interval; 5-FU, 5-fluorouracil; BSC, best supportive care; IV, intravenous.

A total of 2,724 patients (median: 277; range: 185–789) recruited were randomized to receive ICIs. Previous use of ICIs was reported as a universal exclusion criterion across all seven included RCTs. KEYNOTE-061 (12) mandated recruiting patients with PD-L1 CPS ≥1 following an interim review of outcome data. Furthermore, KEYNOTE-062 (13) randomized patients based on PD-L1 CPS ≥1.

Three comparator treatment interventions were analyzed: placebo, best supportive care, and chemotherapy (cisplatin, 5-FU, capecitabine, S-1, irinotecan, oxaliplatin, and paclitaxel). The immune checkpoint inhibitors used were pembrolizumab [two trials (12, 13)], nivolumab [three trials (9–11)], and avelumab [two trials (14, 15)]. Both experimental and comparator arms were administered until disease progression, unacceptable toxicity, or withdrawal of study consent. Most of the included studies (85.7%) had a low overall risk of bias (**Supplementary** **Table 1**; **Supplementary Table 2**).

### Pooled analysis for OS

**Figure 1:**
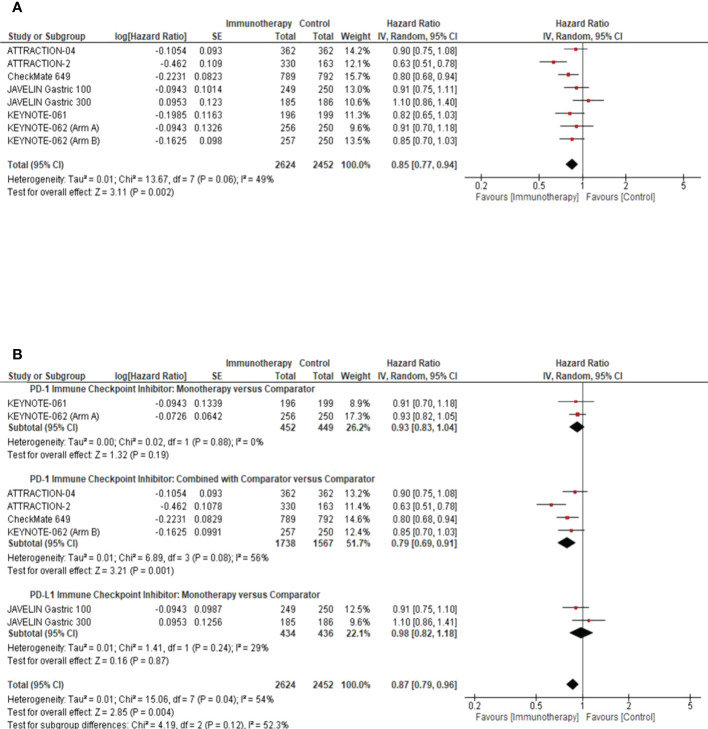
Overall Survival Analysis in the intention to treat population. **(A)** Pooled analysis forest plot to demonstrate the median Overall Survival (OS) in the intention to treat population. **(B)** Subgroup analysis forest plot illustrates the impact of the class of ICI studied, as monotherapy or in combination with the comparator arm, on the median OS of the intention to treat population. Comparator arm: the active treatment in the control arm as defined by the study methods. CI, confidence interval; SE, standard error.

Results of the meta-analysis in the overall study population demonstrated that GC patients randomized to the ICI arm had significantly better OS than patients in the comparator arm (pooled HR: 0.85; 95% CI: 0.77–0.94; *P* = 0.002; *I* ^2^ = 49%) (**Figure 1A**). We performed a subgroup analysis to determine the efficacy of the class of ICI agents. We demonstrated that PD-1 ICIs had an additive effect when combined with the comparator arm among GC patients (pooled HR: 0.79; 95% CI: 0.69–0.91; *P* = 0.001; *I* ^2^ = 56%) (**Figure 1B**).

### Efficacy of ICIs in the Asian patient subpopulation

**Figure 2:**
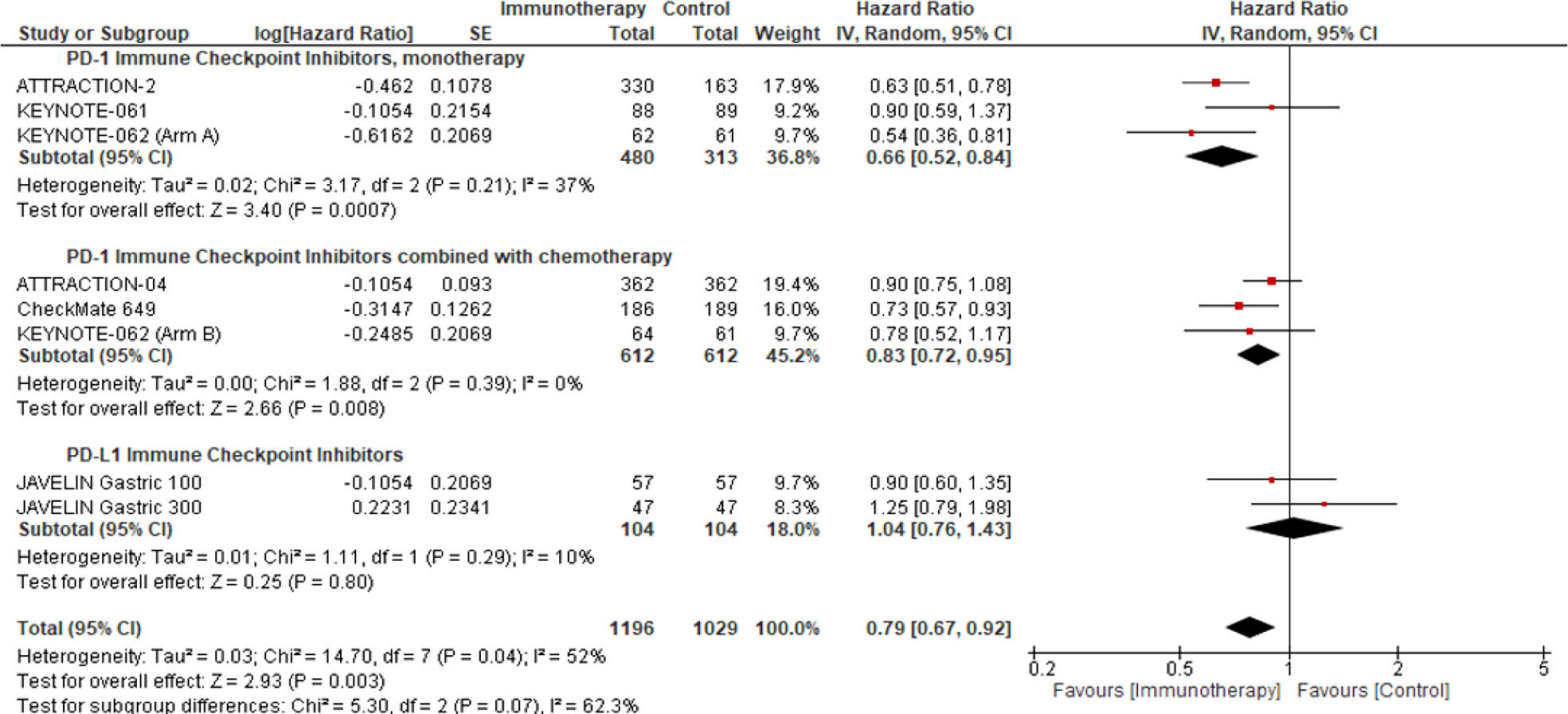
Overall survival response analysis of ICIs in the Asian patient population. This figure illustrates the pre-planned sensitivity analysis forest plot to demonstrate the impact of ICI as monotherapy or in combination with the comparator on the median overall survival in Asian patients’ intention to treat population.

A pre-planned sensitivity analysis was performed to determine the potential impact of the class of ICIs studied on the Asian sub-population. We demonstrated a statistically significant improvement in OS benefit with PD-1 ICIs in the Asian subpopulation, used as monotherapy (pooled HR: 0.66; 95% CI: 0.52–0.84; *P* = 0.0007; *I^2^* = 37%) or in combination with chemotherapy (pooled HR: 0.83; 95% CI: 0.72–0.95; *P* = 0.008; *I^2^* = 0%) (**Figure 2**).

### Association of tissue biomarkers on response to ICIs: Impact of PD-L1 status

**Figure 3:**
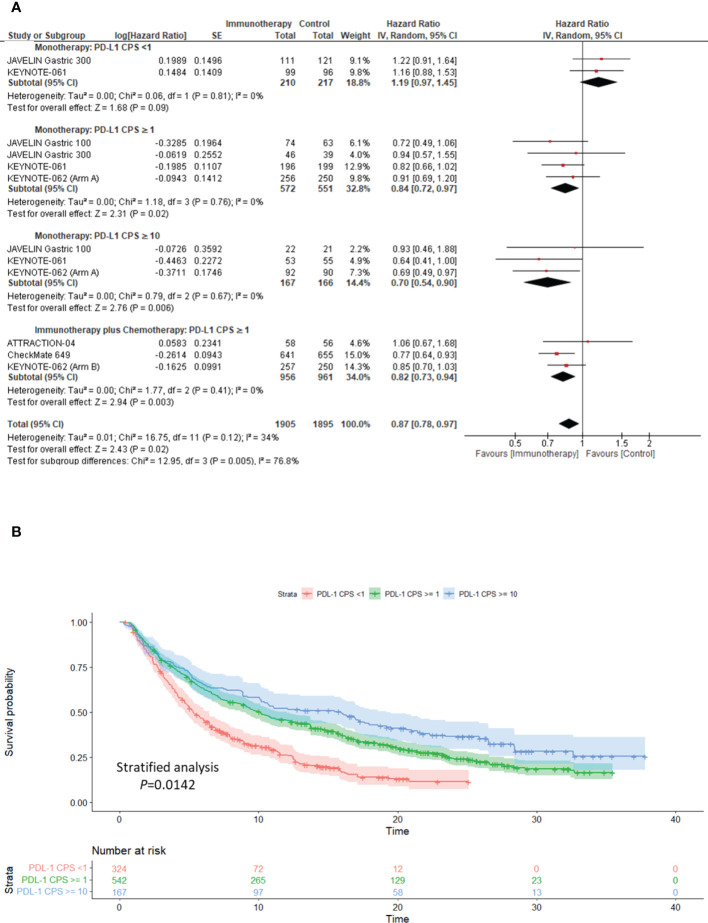
Clinical utility of PD-L1 as a biomarker for response to ICIs in advanced gastric cancer. **(A)** sensitivity analysis forest plot to demonstrate the impact of PD-L1 CPS on Overall Survival following treatment immunotherapy either as monotherapy or in combination with the comparator. **(B)** Kaplan–Meier overall survival curve illustrating the clinical relevance of PD-L1 in determining response to single-agent immunotherapy. PD-L1, programmed cell death ligand-1; CPS, combined positive score; CI, confidence interval.

We performed a pre-planned sensitivity analysis to assess the impact of PD-L1 on the response to ICIs compared to the comparator arm. We report that patients with PD-L1 ≥1 and PD-L1 ≥10 derive OS benefit from ICI monotherapy versus the comparator (PD-L1 ≥1: pooled HR: 0.84; 95% CI: 0.72–0.97; *P* = 0.02; *I^2^* = 0%; PD-L1 ≥10: pooled HR: 0.70; 95% CI: 0.54–0.90; *P* = 0.006; *I^2^* = 0%), with a low level of heterogeneity (**Figure 3A**).

Furthermore, patients with PD-L1 ≥1 who received ICI and chemotherapy showed significantly better OS than those undergoing chemotherapy alone (pooled HR: 0.82; 95% CI: 0.73–0.94; *P* = 0.003; *I^2^* = 0%), with a low level of heterogeneity (**Figure 3A**). In patients with PD-L1 <1, ICIs used as monotherapy versus chemotherapy showed no significant impact on OS (pooled HR: 1.19; 95% CI: 0.97–1.45; *P* = 0.09; *I^2^* = 0%) (**Figure 3A**).

Interestingly, we constructed the time-to-event curve from extracted OS KM curve data in the ICI monotherapy arm. We further demonstrated that GC patients with PD-L1 ≥1 (Stratified analysis *P <*0.0001) and PD-L1 ≥10 (Stratified analysis *P* = 0.0142) derived statistically significant OS benefits to ICI monotherapy when compared to PD-L1 <1 (**Figure 3B**).

### Association of tissue biomarkers on response to ICIs: Impact of MSI-H and TMB status

**Figure 4:**
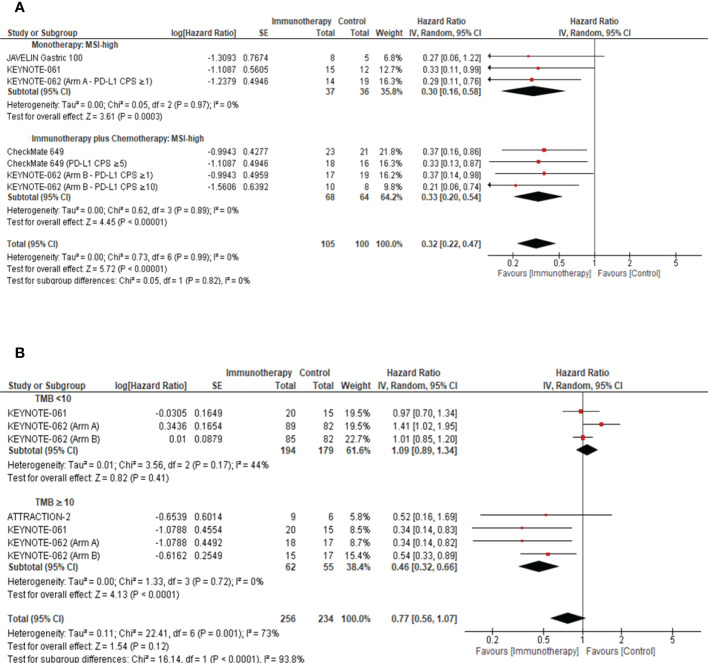
Role of tissue biomarkers on response to ICIs in advanced gastric cancer. **(A)** Forest plot to demonstrate the impact of MSI on Overall Survival in the intention to treat population. **(B)** Forest plot to demonstrate the effect of TMB on the Overall Survival in the intention to treat population. MSI, microsatellite instable; TMB, tumor mutational burden; CI, confidence interval.

In a preplanned sensitivity analysis, we evaluated the roles of both MSI-H and TMB as predictive biomarkers for response to ICIs in GC. Patients with MSI-high tumors derived a significant favorable OS benefit from ICI compared with the chemotherapy arm (ICI monotherapy: pooled HR: 0.30; 95% CI: 0.16–0.58; *P* = 0.0003; *I^2^* = 0%; ICI plus chemotherapy: pooled HR: 0.33; 95% CI: 0.20–0.54; *P <*0.00001; *I^2^* = 0%) (**Figure 4A**). Remarkably, in patients with a TMB ≥10, ICIs were associated with a statistically significant improvement in OS when compared with the comparator (pooled HR: 0.46; 95% CI: 0.32–0.66; *P <*0.0001; *I^2^* = 0%) (**Figure 4B**).

### Safety analysis

All seven RCTs reported grade ≥3 immune treatment-related adverse events (TRAEs). We demonstrated that ICIs did not confer increased TRAEs compared to the control arm (pooled RR: 0.74; 95% CI: 0.42–1.28; *P* = 0.28; *I^2^* = 97%), although there was a presence of substantial heterogeneity (**Supplementary Figure 2**).

## Discussion

ICIs have demonstrated promising OS benefits among advanced GC patients in first-line through third-line settings (25). However, as presented in this study, irrespective of the trend towards enriched survival outcomes with ICIs, it is imperative that we identify the appropriate clinical setting and predictive tissue biomarkers which correlate with response to ICIs. Results presented in this study suggest that MSI-H, TMB-H, and PD-L1 CPS ≥1 tumors should be offered ICIs as they are associated with clinically significant survival outcomes (26). The cut-off for PD-L1 CPS as a biomarker to determine the eligibility of patients for ICI therapy has been controversial. However, there is some consensus in the guidelines that endorse using ICIs in tumors with a PD-L1 CPS≥5 (27, 28). Despite the evidence reported in the CheckMate-649 trial, we could not assess the clinical relevance of the CPS cut-off of≥5 in this current study, primarily because the data for this PD-L1 CPS cut-off sub-group is underpowered and may not adequately reflect the clinical trial observations. Additionally, in the trials included in this meta-analysis, diverse PD-L1 testing methods were employed, namely, Dako 28-8 and Dako 22C3, which added another layer of complexity across all seven trials. Therefore, as ICIs are now becoming the standard of care in advanced GC, if we aim to utilize PD-L1 status as a biomarker for patient selection, there is an unmet need to regulate and harmonize the PD-L1 testing methods globally (29).

Nevertheless, a molecularly stratified approach using predictive immune biomarkers will aid in justifying the exposure of patients to potentially substantial immune-related toxicities and fatalities (30, 31). Interestingly, we highlighted that the immune checkpoint target being pursued plays an important role when interpreting the potential survival impact of ICIs in advanced GC. When combined with the comparator arm, we demonstrated that PD-1 ICIs were associated with improved OS rates (pooled HR: 0.79; 95% CI: 0.69–0.91; *P* = 0.001; *I* ^2^ = 56%) (**Figure 1B**). However, these results must be interpreted with caution given the RCT sample size in each cohort.

We identified that the Asian patient population represented 43.08% of our study cohort and ascertained a potentially clinically meaningful improvement in OS with PD-1 inhibitors (monotherapy: pooled HR: 0.66; 95% CI: 0.52–0.84; *P* = 0.0007; *I^2^* = 37%; combined with chemotherapy: pooled HR: 0.83; 95% CI: 0.72–0.95; *P* = 0.008; *I^2^* = 0%) (**Figure 2**) in this patient population. However, we must interpret these results cautiously since the small sample size may increase the likelihood of type I error. Notably, in the KEYNOTE-062, pembrolizumab monotherapy was associated with preferential survival benefit in the Asian patient subgroup with a PD-L1 CPS ≥1 (HR: 0.54; 95% CI: 0.35–0.82) and PD-L1 CPS ≥10 (HR: 0.43; 95% CI: 0.21–0.89) (32). Conversely, the survival benefit reported in the Asian patient subgroup of KEYNOTE-062 was not observed in KEYNOTE-061 (HR: 0.90; 95% CI: 0.59–1.38) (12).

Irrespective of the patient’s ethnicity, the prognostic significance of PD-L1 CPS status in advanced GC remains controversial. Kim et al. illustrated that patients with PD-L1-positive tumors had a higher ORR (50% versus 0%, P <0.001) than PD-L1-negative tumors to PD-1 inhibition (7). In line with these findings, we have demonstrated an association between PD-L1 expression and response to ICIs. Furthermore, we established that PD-L1 CPS ≥1 and ≥10 enriched for better response to ICIs as monotherapy and in combination with chemotherapy, compared to PD-L1 CPS <1 (**Figure 3**). We demonstrated that ICIs used as monotherapy in patients with PD-L1S CPS <1 had no significant impact on OS compared with chemotherapy (pooled HR: 1.19; 95% CI: 0.97–1.45; *P* = 0.09; **Figure 3A**), although its benefit over best supportive care was shown in salvage line treatment (9). We highlighted that ICIs might exert an additional survival benefit when combined with the comparator arm, such as ATTRACTION-2 (nivolumab with best supportive care) (9), CheckMate-649 (nivolumab plus chemotherapy) (10), ATTRACTION-04 (nivolumab plus chemotherapy) (11), and KEYNOTE-062 (pembrolizumab plus chemotherapy) (13). A head-to-head comparison between ICI monotherapy and the comparator arm did not demonstrate a substantial OS benefit in the intention-to-treat group (**Figure 1B**).

The incidence of TMB-high and MSI-H tumors in our study cohort was 2.7% and 4.7%, respectively. Irrespective of the small sample size, data presented in our systematic review suggested that the subgroup of advanced GC with an MSI-H (Monotherapy: *P* = 0.0003, Combination of ICI and chemotherapy: *P <*0.00001, **Figure 4A**) or TMB-high (TMB ≥10) (*P <*0.0001, **Figure 4B**) genomic profile demonstrated better OS outcomes. The tumor mutational load was previously reported as an independent predictor for response to ICIs (33). Wang et al. (34) demonstrated that advanced GC classified as PD-L1-positive and TMB-high were associated with superior PFS (HR: 0.45; 95% CI: 0.24–0.85; *P* = 0.014) and OS (HR: 0.47; 95% CI: 0.24–0.92; *P* = 0.027) when compared to PD-L1-negative and TMB-low tumors (34). Yu et al. demonstrated that in NSCLC, TMB-high or PD-L1-high tumors were associated with better response rates (62.5%) and durable clinical benefit (77.3%) (35). It is important to note that neither trial was statistically powered to assess the predictive value of TMB and MSI status on PD-L1 expression. However, these outcomes were consistent with findings in other solid tumors, which led to the accelerated FDA approval of pembrolizumab and nivolumab for patients with MSI-H/mismatch repair (MMR)-deficient (dMMR) metastatic solid cancer (36). Furthermore, Kim et al. demonstrated a positive correlation between TMB and pembrolizumab response (AUC: 0.74; *P* = 0.006) (7). Our findings were consistent with those reported by Pietrantonio et al. (37), which illustrated that the MSI-H subgroup conferred OS benefit from ICIs compared to chemotherapy (HR: 0.34; 95% CI: 0.21–0.54; *P* = 0.003) (37). The findings reported by Shitara et al. demonstrated that whole-exome sequencing (WES) of tumoral TMB (tTMB) was associated with favorable clinical outcomes in pembrolizumab-treated patients (one-sided p: <0.001) (38). Importantly, the clinical benefit of pembrolizumab persisted in microsatellite stable (MSS) WES tTMB-high (≥175 mut/exome) (38). We advocate for a novel, stratified trial design incorporating TMB and MSI for patient stratification to harness a precision oncology approach in advanced gastric cancer and to explore their predictive role in ICI response.

We conducted a literature search to identify whether any relevant meta-analysis in the setting of advanced GC had been published prior to reporting this study and identified six studies of interest (**Supplementary Table 3**) (37, 39–43). However, we have identified heterogeneity in the results across the six studies. The inconsistencies in these studies may be explained by insufficient data available at the time of publication. The data reported in our meta-analysis is the most comprehensive. It incorporates data from all the published clinical trials that have shifted the treatment paradigm for advanced GC. Our study has incorporated a novel survival curve reconstruction to emphasize the impact of PD-L1 status on survival (24, 44). In addition, we performed a thorough analysis to demonstrate the survival impact of tumoral PD-L1 expression, TMB, and MSI on response to immune checkpoint inhibitors.

Nonetheless, although the role and clinical indications of ICIs in advanced GC are rapidly evolving, we have identified some significant limitations to this study. Firstly, this study is limited by the paucity of clinical data to comprehensively explore the conclusive impact of TMB on OS outcomes in advanced GC following treatment with immune checkpoint inhibitors. A collaborative approach combining MSI data and TMB across all clinical trials would offer a more meaningful assessment of MSI-H and TMB in GC. Secondly, given the relatively limited number of randomized ICI trials conducted among treatment refractory and treatment naïve GC patients, we need to be cautious when inferring these observations. Of importance, the pooled OS of all included trials in this study reported a heterogeneity score of *I* ^2^ = 49% (p = 0.06), which indicates there was moderate variability. Hence, as the number of randomized control ICI trials continues to expand in advanced GC, we propose that there should be a follow-up meta-analysis to continually monitor the relevance and clinical efficacy of ICIs across the treatment paradigm of advanced gastric cancer.

In conclusion, this study indicates that ICIs may be essential in managing advanced GC. In particular, clinical efficacy of ICIs was observed in PD-L1-positive, MSI-H, and TMB-high advanced GC. Thus, our data and the clinical studies described above collectively support the rationale for routine testing for MSI, TMB, and PD-L1 status in all newly diagnosed locally advanced, unresectable, or metastatic GC.

## Data availability statement

The original contributions presented in the study are included in the article/**Supplementary Material**. Further inquiries can be directed to the corresponding author.

## Author contributions

Conceptualization: AE. Methodology: AE, JT, CW, and HP. Formal analysis: AE, JT, CW, and HP. Writing—original draft: AE. Writing—review and editing: AE, CW, W-LC, K-CM, WW, MM, KS, NB, HP, and KL. All authors contributed to the article and approved the submitted version.

## Conflict of interest

AE disclosure: Advisory role: Pfizer, Bayer, Almac Discovery, Almac Diagnostics Research funding: Almac Diagnostics. MM disclosure: Honoraria: Taiho Pharmaceutical, Eli Lilly/ImClone, Amgen, Roche/Genentech, Merck KGaA, Darmstadt, Germany, MSD Oncology, Bristol Myers Squibb, AstraZeneca/MedImmune, Servier Consulting or Advisory Role: Bayer, Merck Sharp & Dohme, Merck KGaA, Darmstadt, Germany, Amgen, Taiho Pharmaceutical, Nordic Group, Pfizer, Yakult, Roche, Eli Lilly, Servier Research Funding: Amgen Inst, Leap Therapeutics Inst, Merck KGaA, Darmstadt, Germany Inst, Jennerex Inst, AstraZeneca Inst, Merck Sharp & Dohme Inst Travel, Accommodations, Expenses: Amgen, Merck KGaA, Darmstadt, Germany, Roche, Bayer, American Society of Clinical Oncology, German Cancer Society, Merck Sharp & Dohme, European Society for Medical Oncology Kohei Shitara disclosure: Honoraria—Abbvie; Novartis; Yakult Pharmaceutical Consulting or Advisory Role—Abbvie; Amgen; Astellas Pharma; Boehringer Ingelheim; Bristol-Myers Squibb; Daiichi Sankyo; GlaxoSmithKline; Lilly; MSD; Novartis; Ono Pharmaceutical; Pfizer; Taiho Pharmaceutical; Takeda; Novartis; Merck Pharmaceutical Research Funding—Astellas Pharma Inst; Chugai Pharma Inst; Daiichi Sankyo Inst; Dainippon Sumitomo Pharma Inst; Eisai Inst; Lilly Inst; Mediscience Planning Inst; MSD Inst; Ono Pharmaceutical Inst; Taiho Pharmaceutical Inst; Merck Pharmaceutical. NB disclosure: Honoraria—Bristol-Myers Squibb Japan; Ono Pharmaceutical; Taiho Pharmaceutical Research Funding—Ono Pharmaceutical Inst; Takeda Inst. HP disclosure: Personal fees from Genentech outside the submitted work. KL disclosure: Honoraria: Eli Lilly; Bristol-Myers Squibb; Daiichi Sankyo; Taiho Pharmaceutical; Merck Pharmaceutical; Amgen; BAYER; MSD; Sanofi-Aventis Consulting or Advisory Role: Eli Lilly; Bristol-Myers Squibb; Daiichi Sankyo; Taiho Pharmaceutical; Merck Pharmaceutical; Amgen; Bayer; MSD Research Funding: Taiho Pharmaceutical; Bayer; Roche.

The remaining authors declare that the research was conducted in the absence of any commercial or financial relationships that could be construed as a potential conflict of interest.

Reviewer SM declared a shared affiliation with the author KS to the handling editor at the time of review.

## Publisher’s note

All claims expressed in this article are solely those of the authors and do not necessarily represent those of their affiliated organizations, or those of the publisher, the editors and the reviewers. Any product that may be evaluated in this article, or claim that may be made by its manufacturer, is not guaranteed or endorsed by the publisher.
