# Supplementary material for: A meta-analysis with systematic review: Efficacy and safety of immune checkpoint inhibitors in patients with advanced gastric cancer
Source: Front Oncol. 2022 Oct 31;12:908026. doi: 10.3389/fonc.2022.908026 (PMC9660259; doi:10.3389/fonc.2022.908026)
Supplement: Supplementary file 1 [file DataSheet_1.docx]

Supplementary methods:

**Literature Search Strategy**

We completed a comprehensive data-mining exercise in the following databases: PubMed, the Cochrane Library, Embase, ClinicalTrials.gov, World Health Organization (WHO) International Clinical Trials Registry Platform (ICTRP), EORTC database of clinical trials, Web of Science Core Collection published before the 1^st^ of October 2020. The American Society of Clinical Oncology (ASCO) annual meeting abstracts, the American Association for Cancer Research (AACR) annual meeting abstracts, and the European Society of Medical Oncology (ESMO) abstracts published before the 1^st^ of October 2020. The key search terms used were "stomach neoplasm” "gastric neoplasm", "stomach cancer", "immunotherapy", "immunotherapies", "checkpoint inhibitors", "PD-1” "PD-L1” "programmed death 1", "programmed death-ligand 1", "programmed cell death ligand 1", "CTLA4", "cytotoxic T-lymphocyte-associated protein 4", "IDO", "Indoleamine-pyrrole 2,3-dioxygenase", "Asia", "Asian Continental Ancestry Group", "pembrolizumab", "nivolumab", "ipilimumab", "atezolizumab", "avelumab", "durvalumab", "cemiplimumab", "epacadostat", "tremelimumab".

Randomized clinical trials (RCT) comparing the efficacy of immune checkpoint inhibitors (ICIs) as monotherapy or in combination with standard of care treatment, as defined in current, evidence‐based guidelines for systemic therapy, in patients with either locally advanced or metastatic gastric cancer, were included in the literature search. The trials must include an Asian patient cohort. Consequently, we excluded studies if they did not provide enough data to obtain survival hazard ratios (HRs).

**Data collection and assessment of study quality**

***Selection of studies***

After a comprehensive literature search, we used reference management software (EndNote) to identify and remove potential duplicate records. Three review authors (AE, KOL, WLC) independently scanned the abstract or title, or both, of remaining records retrieved to further assess which studies should be evaluated. Three review authors (AE, KOL) investigated all potentially relevant records as full text, mapped records to studies, and classified studies as included studies, excluded studies, studies awaiting classification, or ongoing studies in accordance with the criteria in the *Cochrane Handbook for Systematic Reviews of Interventions*^1^. We resolved any discrepancies through consensus or recourse to a third review author (WLC). If a disagreement was not possible, we designated the study as 'awaiting classification' and contacted trial authors for clarification. We presented an adapted PRISMA flow diagram showing the process of study selection^2^.

***Data extraction***

For trials that fulfilled inclusion criteria, one review author (AE) abstracted the following information from individual studies, which were provided in the characteristics of included studies tables: study design, study dates, study settings, and country; participant inclusion and exclusion criteria; participant details, baseline demographics; the number of participants by study and by study arm; details of relevant experimental and comparator interventions such as dose, route, frequency, and duration; definitions of relevant outcomes, and method and timing of outcome measurement as well as any relevant subgroups; and study funding sources. In addition, we defined the following endpoints as subjective outcomes: quality of life (QoL), immunotherapy-related adverse events; overall survival (OS); progression-free survival (PFS), and objective response rates (ORR). Data extracted was further independently evaluated by two authors (KOL, WLC). We defined the comparator as the active treatment in the control arm or delivered in combination with immunotherapy.

***Duplicate and companion publications***

In the event of duplicate publications, we gave priority to the publication reporting the most extended follow‐up associated with our primary and secondary outcomes.

***Assessment of risk of bias***

Two review authors (AE, KOL) assessed the risk of bias of each included study independently. We resolved disagreements by consensus or consultation with a third review author (WLC). We evaluated the risk of bias using Cochrane's 'Risk of bias' assessment tool^3^. The following domains were assessed; random sequence generation (selection bias); allocation concealment (selection bias); blinding of participants and personnel (performance bias); blinding of outcome assessment (detection bias); incomplete outcome data (attrition bias); selective reporting (reporting bias); and other sources of bias. We presented a Risk of bias summary figure to illustrate these findings further*.* Blinding was not performed in all trials, but the assessment of survival was not likely to be influenced by lack of blinding.

References:

1. Higgins, J. P. T. & Green, S. Cochrane Handbook for Systematic Reviews of Interventions Version 5.1.0 [updated March 2011]. in *The Cochrane Collaboration* (2011).

2. Moher D Tetzlaff J, Altman Dg, L. A. PRISMA 2009 Flow Diagram. *The PRISMA statement* (2009).

3. Higgins, J., Savović, J., Page, M. J. & Sterne, J. A. C. RoB 2: A revised Cochrane risk-of-bias tool for randomized trials. *British Medical Journal* (2019).
