# Supplementary material for: A meta-analysis with systematic review: Efficacy and safety of immune checkpoint inhibitors in patients with advanced gastric cancer
Source: Front Oncol. 2022 Oct 31;12:908026. doi: 10.3389/fonc.2022.908026 (PMC9660259; doi:10.3389/fonc.2022.908026)
Supplement: Supplementary file 2 [file Presentation_1.pptx]

## Slide 1
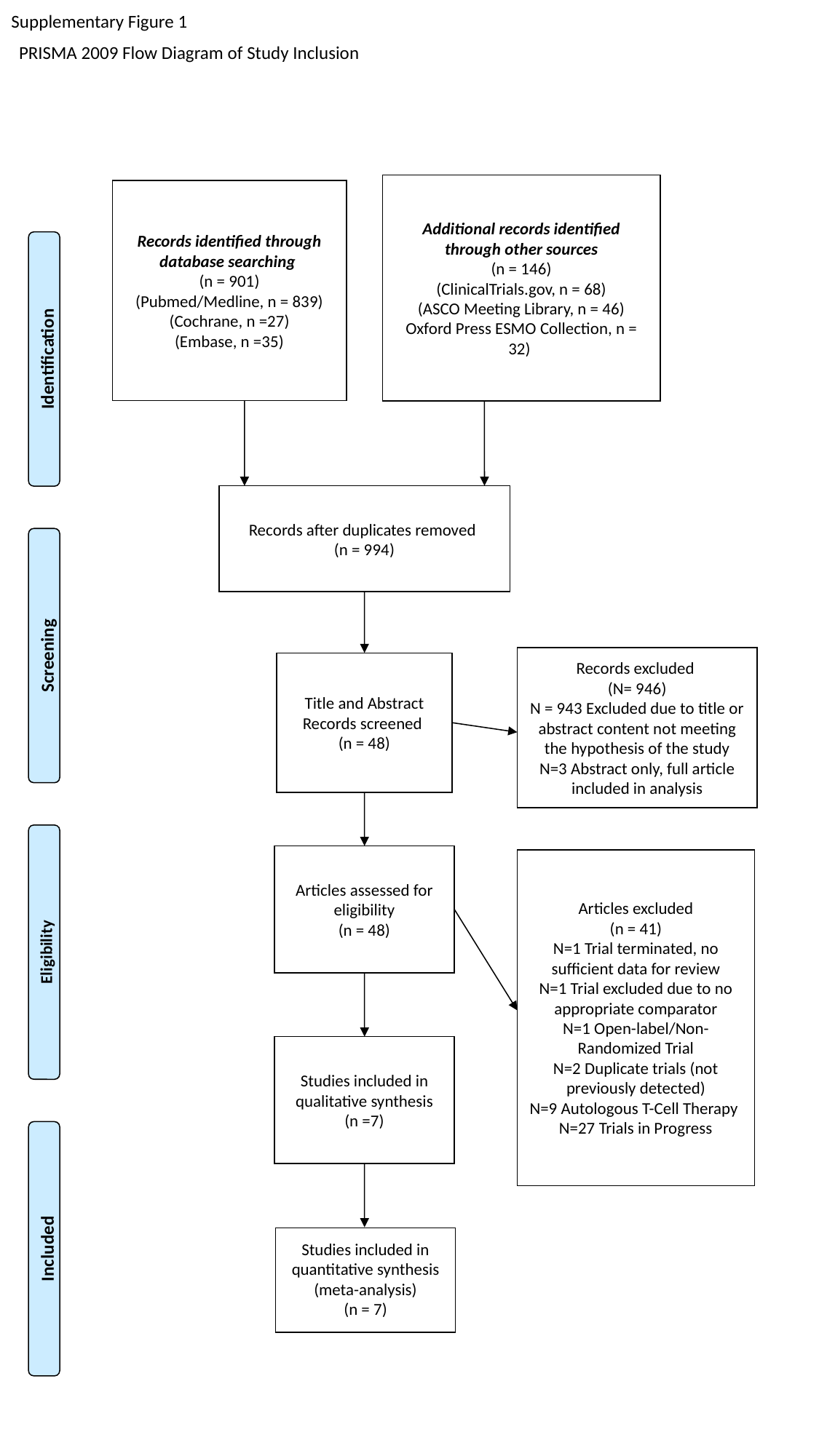

Supplementary Figure 1
PRISMA 2009 Flow Diagram of Study Inclusion
Additional records identified through other sources
(n = 146)
(ClinicalTrials.gov, n = 68)
(ASCO Meeting Library, n = 46)
Oxford Press ESMO Collection, n = 32)
Records identified through database searching
(n = 901)
(Pubmed/Medline, n = 839)
(Cochrane, n =27)
(Embase, n =35)
Identification
Records after duplicates removed
(n = 994)
Screening
Records excluded
(N= 946)
N = 943 Excluded due to title or abstract content not meeting the hypothesis of the study
N=3 Abstract only, full article included in analysis
Title and Abstract Records screened
(n = 48)
Articles assessed for eligibility
(n = 48)
Articles excluded
(n = 41)
N=1 Trial terminated, no sufficient data for review
N=1 Trial excluded due to no appropriate comparator
N=1 Open-label/Non-Randomized Trial
N=2 Duplicate trials (not previously detected)
N=9 Autologous T-Cell Therapy
N=27 Trials in Progress
Eligibility
Studies included in qualitative synthesis
(n =7)
Included
Studies included in quantitative synthesis (meta-analysis)
(n = 7)
