# Supplementary material for: A meta-analysis with systematic review: Efficacy and safety of immune checkpoint inhibitors in patients with advanced gastric cancer
Source: Front Oncol. 2022 Oct 31;12:908026. doi: 10.3389/fonc.2022.908026 (PMC9660259; doi:10.3389/fonc.2022.908026)
Supplement: Supplementary file 3 [file Presentation_2.pptx]

## Slide 1
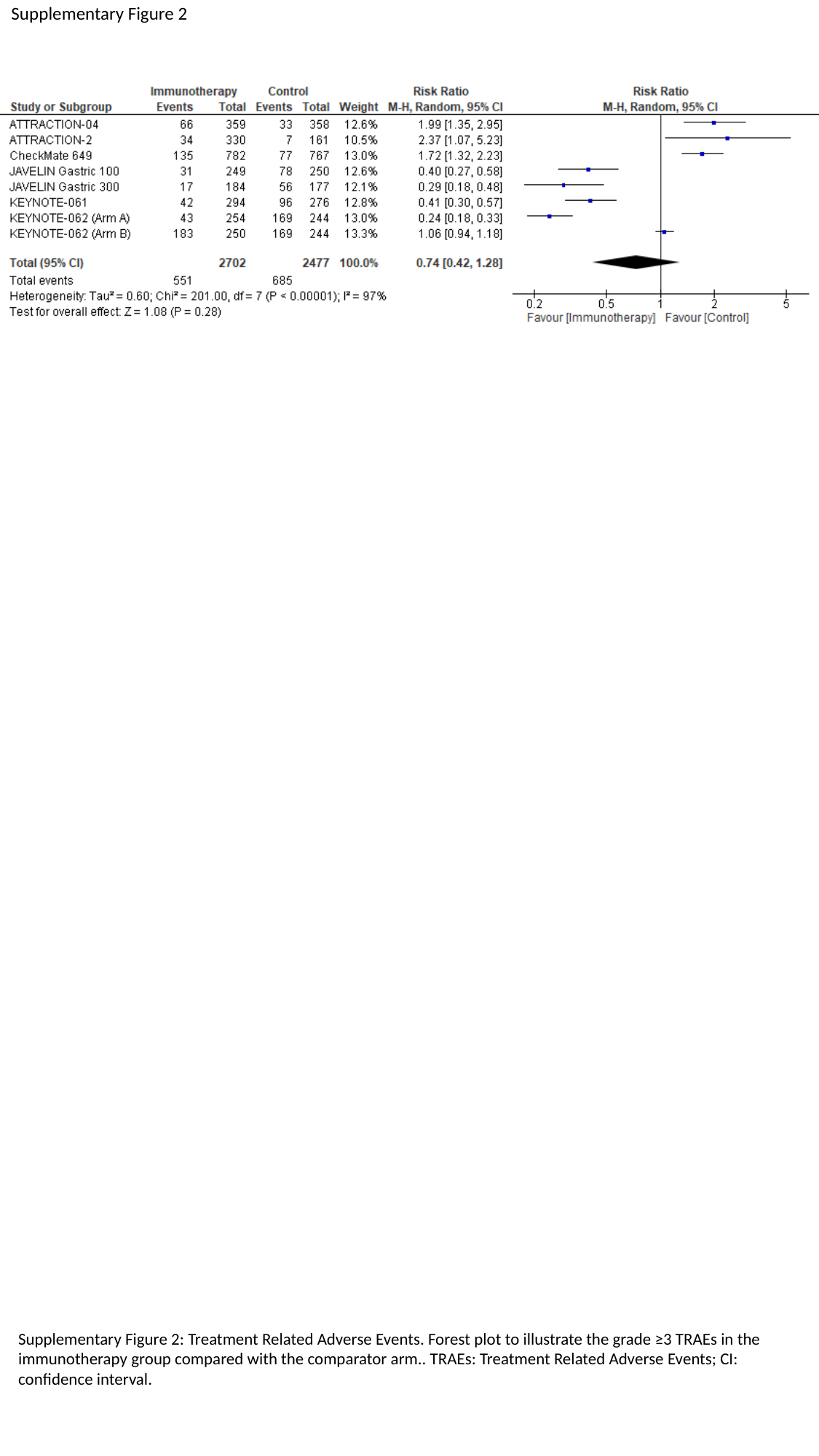

Supplementary Figure 2
Supplementary Figure 2: Treatment Related Adverse Events. Forest plot to illustrate the grade ≥3 TRAEs in the immunotherapy group compared with the comparator arm.. TRAEs: Treatment Related Adverse Events; CI: confidence interval.
